# Meeting the Consultation Surge: A Nationwide Survey of Consult Volume and Mitigation Strategies in Infectious Diseases Fellowship Programs

**DOI:** 10.1093/ofid/ofae123

**Published:** 2024-03-05

**Authors:** Alfredo J Mena Lora, Ryan Knodle, Scott Borgetti, Brionna Matt, Georgina Osorio, Vidya Sundareshan, Christian Rojas-Moreno, Rachel Bartash, Trevor C Van Schooneveld, Rebecca Reece, Brian Chow, Katya Prakash, Saira Butt, Allison Lastinger

**Affiliations:** Department of Medicine, Division of Infectious Diseases, University of Illinois at Chicago, Chicago, Illinois, USA; Department of Medicine, Division of Infectious Diseases, University of Illinois at Chicago, Chicago, Illinois, USA; Department of Medicine, Division of Infectious Diseases, University of Illinois at Chicago, Chicago, Illinois, USA; Lewis Katz School of Medicine at Temple University, Philadelphia, PA, USA; Icahn School of Medicine at Mount Sinai, New York, NY, USA; Southern Illinois University School of Medicine, Carbondale, IL, USA; Division of Infectious Diseases, University of Missouri-Columbia, Columbia, IL, USA; Division of Infectious Diseases, Montefiore Medical Center/Albert Einstein College of Medicine, New York, NY, USA; Division of Infectious Diseases, University of Nebraska Medical Center, Omaha, NE, USA; Albert Einstein College of Medicine, New York, NY, USA; Division of Geographic Medicine and Infectious Diseases, Tufts Medical Center, Boston, Massachusetts, USA; University of Maryland School of Medicine, Baltimore, MA, USA; Division of Infectious Diseases, Indiana University School of Medicine, Indianapolis, Indiana, USA; Division of Infectious Diseases, West Virginia University School of Medicine, Morgantown, WV, USA

**Keywords:** caps, high volume, medical education, workload, fellowship training

## Abstract

High patient volume in fellowship programs can affect learning, wellness, and patient outcomes. Training programs must find ways to mitigate high consultation volume to protect the learning environment. This survey describes average new consults and average censuses for infectious diseases training programs and strategies implemented to mitigate high volume.

## Teaser

High patient volumes in training programs can impact learning, wellness, and patient outcomes. Mitigating high volume is imperative to protect the learning environment. This nationwide survey describes the average workload for infectious diseases fellows and mitigation strategies implemented by programs.

During the 2022 IDWeek Program Director's meeting, an increase in inpatient infectious diseases (ID) consult volume was identified as an area of concern [1]. The balance between delivering high-quality patient care, maintaining a reasonable patient census, and nurturing the educational experience of trainees is a priority for program directors [1, 2]. The Accreditation Council for Graduate Medical Education has provided patient census and admissions recommendations for internal medicine residency programs since 1980, which evolved to specific cap requirements that have been strengthened over the years. However, there are no such regulations for ID subspecialty training programs [3].

During the first 2 years of the coronavirus disease 2019 pandemic, a surge of ID fellowship applications occurred, with 404 and 387 applicants in the 2021 and 2022 match cycles, respectively [4]. However, a significant decline in ID program applications has occurred since, with only 56% of programs filling their slots for the appointment year 2023 and 51% for 2024 [5, 6]. The recruitment struggles in ID fellowships are likely multifactorial. However, addressing consult workload and its potential ramifications on the learning environment, wellness, and burnout for current fellows may be important for recruitment of future fellows [7–9]. We performed a survey of ID fellowship programs in the United States to assess consult volume trends and mitigation strategies implemented by fellowship programs in the United States.

## METHODS

At the 2022 IDWeek program director's meeting, a breakout session was dedicated to managing volume with time for education during inpatient rounds. As a follow-up to this session, a web-based survey to quantify consult volumes in ID fellowship programs in the United States was developed in Microsoft Forms to capture data on characteristics of fellowship programs, number of first-year fellows, total number of fellows, total number of faculty, type of rotations, type of hospital, and number hospital beds. On 22 and 25 April and 19 October 2023, we distributed the survey via online messaging boards and listservs for members of the Infectious Diseases Society of America (IDSA) training program directors. The survey reflected the academic year 2022–2023. All responses were submitted anonymously. We surveyed characteristics of the self-reported busiest rotation in respondents’ fellowship programs, including the volume of new consults per day, total consults followed daily per fellow, and type of rotations. Data on mitigation strategies was collected, including caps for new consults or total consults, caps for new or total consults per fellow, and the strategies used by programs once caps are met (Appendix 1).

Informed consent was implied through participant engagement. Information about the survey’s purpose and the voluntary nature was provided before survey commencement, with continuation into the survey serving as consent. No personal identifying information or protected information was collected, ensuring participant anonymity. Participation was voluntary, anonymous and without financial incentives. Our study was reviewed by the University of Illinois Institutional Review Board.

## RESULTS

### Program and Rotation Description

There were a total of 33 responses, corresponding to a 19% response rate from 178 programs nationwide. The average time to complete the survey was 6 minutes 34 seconds. Programs and rotation characteristics are described in Table 1.

**Table 1.** Program Characteristics

Survey Question	Response
What is the practice setting for your fellowship program?	University hospital, 30; community hospital, 5; university-affiliated community hospital, 5; VA hospital, 5; county hospital, 2; other, 0
Where is the busiest inpatient fellowship rotation in your program located**?**	University hospital, 28; community hospital, 1; university-affiliated community hospital, 3; VA hospital, 0; county hospital, 1; other, 0
What is the size of this hospital?	<200 beds, 0; 201–300 beds, 1; 301–400 beds, 3; 401–500 beds, 4; >500 beds, 25
How many first-year fellow positions do you have?	Average, 3.69; range, 2–10
What is the total number of fellows in your program?	Average, 7.69; range, 2–20
What is the total number of clinical faculty in your program?	Average, 22.84; range, 6–100
What best describes the rotation or patient population on your busiest rotation?	General ID, 21; general ID and immunocompromised team, 7; immunocompromised team, 5
In your busiest rotation, please select or type the structure of this rotation	1 team with 1 fellow and 1 attending, 15; 1 team with 2 fellows and 1 attending, 3; 2 teams with 1 fellow and 1 attending covering each team, 13; 3 teams with 1 fellow and 1 attending covering each team, 2

Abbreviations: ID, infectious diseases; VA, Veterans Administration.

### Census and Mitigation

The average number of new consults that fellows were responsible was 5.5 per day (range, 3–12). A cap on new consults was in place in 10 programs (30%), with an average cap of 5.3 (range, 4–7). Once the new consult cap was reached, programs used the following mitigation strategies: coverage of consults by faculty (25 of 33 programs) and use of additional fellows, advanced practice providers, and overflow to another team (1 program each). Use of nonteaching services was the most common strategy to help mitigate high census and was used by 19 programs (58%). The average total census that a fellow was responsible for per day (new consults and follow-ups, including those seen by others in the team, such as medical students or residents) was 17.06 (range, 8–30). A cap on the total census was used by only 5 programs (15%), and the average cap was 18 (range, 8–22). When the total census cap is reached, mitigation strategies included coverage of consults by faculty (10 programs), advanced practice providers (1 program), and overflow to another team (2 programs). Eighteen programs provided additional free-text comments (Table 2).

**Table 2.** Excerpts From Survey Responses on Mitigation Strategies Implemented by Programs

Subject	Responses
Overflow or offloading to faculty (faculty on the primary service)	“Faculty on the team … step in. The consults are then alternated, 2 by faculty and 2 by fellow.” “We depend on faculty helping out. Attendings will help with seeing new consults and writing follow-up notes.” “The weekends can be rough with 1 fellow on and about 5–10 consults a day. It is essential for the attending to help here.” “Extensive help from attendings if volumes are high.” “When we have 1 attending and 1 fellow in the team, we established a cap for new consults and total census. The attending on service takes on cases when the caps are reached.” “Fellows cover 2 hospitals on the weekends. If we reach the cap on our busy hospital, faculty is responsible for patients on the other hospital and fellows no longer have to round there.”
Overflow or offloading to fellows	“We manage volume with intensive collaboration between teams.” “When the cap is reached, fellows are pulled from services and rotations that are less busy.”
Overflow or offloading to advanced practice providers	“2 APN services with attending support are taking increased numbers of patients.” “Use of PAs has helped a lot [with increased volume].” “We have our NP see patient[s] who are on a stable antibiotic plan with no further ID diagnostics planned (inpatient).” “We have addressed rising census/consults by adding the services of an inpatient PA who works with the ID fellow and 1 ID attending (+ any medical students, residents who are rotating on ID).”
Additional team	“We have added 2 nonteaching services and an OPAT service managed by [the] Antibiotic Stewardship [team].” “When general ID census [surpasses] >20, some new consults are diverted to ortho ID team. Plus have a follow-up service which picks up patients after general ID signs off and if very busy may hand off patients a little earlier than usual.” “Having specialized services (transplant ID) had an impact [on increasing volume].” “At our busiest site, we have an attending-only general ID service that helps to handle overflow on busy days.” “We are considering a third service with faculty only to help with 2–3 consults each day to minimize the number of new consults on teaching team.” “We have a busy general ID service which when over 20 patients may overflow 1–2 new consults per weekday to our ortho ID service (1 attending, 2 APPs) as long as it is adequately staffed.” “A follow-up service (attending alone) which picks up patients after general ID has signed off and can offload some of the general ID service at times.” “Send overflow consults to transplant ID service.” “Second team of faculty/PA see 4 new consults and primary teaching team of faculty/fellow/resident team sees the others.” “[Total] census split [between] teaching and nonteaching service.” “We created an attending-only consult service for the general ID team.” “We have paired teaching and nonteaching teams [to mitigate volume].” “We expanded our transplant ID service from 1 service to 2 solid organ transplant ID and 1 BMT/Onc ID team.”
Adding FTE/recruitment	“We advocated and provided documentation (consult logs over a year and the increase over last 3 [years]) to the hospital … to fund another fellow line.” “We added an additional fellow this academic year and now have a team with 2 fellows and 1 faculty. Fellows divide consults evenly.” “We argued that staffing the transplant service to be able to do [all necessary] consults … at the speed they needed required an additional fellow.”
Faculty triage and curbsides	“Attendings take curbsides to lessen the burden on fellows [which] … does help decrease the number of consults we do as many do not need to be full consults.” “We have an attending hold the new consult triage pager from 8–5 on weekdays, which has helped a lot in terms of workload (this pager receives all questions, curbsides, and new consult requests).” “We have more issues on the weekends when there are fewer people around to see consults. Very often attending and fellow triage to cancel or give curbside advice.” “Attending [addresses curbside questions] attending to determine … inpatient consult versus outpatient versus e-consult.”
Pharmacy solutions	“We have an antibiotic approval system run through pharmacy so fellows do not have to field those calls.”
Residents and other learners	“Residents help with [consults and] notes.” “Multiple additional learners [help with consults].” “The fellow is not responsible for the patients seen by other learners who include other non-ID fellows, residents, students etc.” “We reached out to addiction medicine, podiatry, orthopedics, and neurosurgery to increase learners [on] our team and help mitigate high volume.” “We created an elective for physician assistant students to rotate with us and increase students on the team.”
Pager solutions	“We have also placed verbiage on their pagers so that when teams start a page to them, they see that there may be other people they should call first.”

Abbreviations: BMT/Onc, bone marrow transplant/oncology; FTE, full time equivalent; ID, infectious diseases; NP, nurse practitioner; PA, physician assistant; OPAT, outpatient parenteral antibiotic therapy.

## DISCUSSION

Our survey describes the census volume and mitigation strategies implemented by ID programs across the country. Safeguarding the educational experience of our fellows by implementing effective strategies to address high consultation volumes is paramount. Studies have shown that high patient census can compromise patient safety, time for education, trainee satisfaction, and documentation while increasing healthcare costs, underscoring the importance of workload management in healthcare settings [2]. Thus, the impact of high volume on program effectiveness and morale could also negatively affect fellow recruitment. At a time when unfilled programs are at a record high, optimizing the workload of fellows to promote wellness and protect the learning environment while optimizing patient care is not just desirable but imperative.

Programs reported daily new consults and total census as high as 12 and 30, respectively, and shared different mitigation strategies including volume caps, hiring more personnel such as nurses, or expanding fellowship positions. The implementation of caps on both new consults and total census emerged as a common mitigation approach, with variations in cap thresholds. When caps were reached, programs relied on a combination of strategies, including seeking assistance from faculty, fellows, and nurse practitioners or transferring overflow cases to other teams. These strategies are vital for managing the high caseload during busy rotations. However, it is essential to recognize their potential downstream effects, such as overburdening ID faculty at a time when faculty burnout is a significant concern or overburdening fellows during less intense rotations or when they are off service [1, 4, 7–9]. These strategies could contribute to further burnout and may indirectly affect faculty and fellow recruitment.

As we navigate the challenges posed by the consultation surge, it is crucial to strike a balance between ensuring quality patient care, preserving educational opportunities, and safeguarding the well-being of both faculty and fellows. This survey may help program directors by sharing the average census from other programs and mitigation strategies that may be adapted in their programs. Further research and ongoing evaluation of mitigation strategies are needed.

Our study has many limitations, including its low response rate and possible selective bias when using the IDSA training directors’ listserv. Most programs responding were university-affiliated sites. Nevertheless, our article presents findings that may shed light on average fellowship workload and the diverse strategies used to protect the learner's environment.
